# Prognostic significance of systemic immune inflammation index and systemic inflammation response index for cervical cancer: A systematic review and meta-analysis

**DOI:** 10.12669/pjms.41.11.12852

**Published:** 2025-11

**Authors:** Feifei Jiang, Peihong Zhou

**Affiliations:** 1 Feifei Jiang, Department of Gynecology, Huzhou Maternity & Child Health Care Hospital, Huzhou, Zhejiang Province 313000, P.R. China; 2 Peihong Zhou, Department of Gynecology, Huzhou Maternity & Child Health Care Hospital, Huzhou, Zhejiang Province 313000, P.R. China

**Keywords:** Malignancy, Females, Survival, Recurrence, Biomarkers, Inflammation

## Abstract

**Objective:**

We present the first systematic review and meta-analysis examining the association between systemic immune inflammation index (SII) and systemic inflammation response index (SIRI) for predicting prognosis of cervical cancer.

**Methodology:**

Cochrane CENTRAL library, Embase, Medline (PubMed), Scopus and Web of Science databases were explored up to 25^th^ October 2024 for all study types examining if SII or SIRI could predict overall survival (OS) and/or disease-free survival (DFS) in cervical cancer.

**Results:**

We included 13 studies with 15 cohorts. We noted that high SII scores were associated with statistically significant increased risk of poor OS (HR: 2.15 95% CI: 1.48, 3.14 I^2^=78%) and worse DFS (HR: 2.05 95% CI: 1.21, 3.46 I^2^=83%) in cervical cancer. Meta-analysis also showed a statistically significant association between SIRI and OS (HR: 1.47 95% CI: 1.02, 2.13 I^2^=67%) but not for DFS (HR: 1.59 95% CI: 0.96, 2.64 I^2^=79%). Subgroup analysis based on country of the study, sample size, cancer stage, treatment, cut-off, use of adjusted data and study quality showed variables results.

**Conclusions:**

SII can be used to predict OS and DFS in cervical cancer. However, SIRI was found to predict only OS and not DFS with less robust results. Further studies shall provide better evidence.

***Registration No:*** PROSPERO: CRD42024603478.

## INTRODUCTION

Cervical cancer ranks the 6^th^ most cancer in females around the world.^1^ The commonest cause is human papillomavirus (HPV) wherein high-risk variants of the virus cause cervical intraepithelial lesions which gradually progress to cancer.^2^ The United States Preventive Services Task Force recommends that a Papanicolaou (Pap) test every three years or both HPV and Pap tests every five years can help prevention in women as young as 21 years.^3^ Furthermore, a vaccine is also available for HPV which can significantly decrease the risk of cervical cancer.^4^ In diagnosed cases, surgery, radiotherapy, chemotherapy and targeted therapies can be used for management depending on the stage of the disease.^5^ Survival can depend upon several factors like cancer stage, lymph node involvement, age, race, ethnicity, comorbidities and treatment protocols.^6^ For early stage cancer, the 5-year survival rate is high at 91% but can be as low as 19% with distant metastasis.^7^ Given the high prevalence and variable survival rates of cervical cancer, there is a need for reliable markers which can accurately predict prognosis.

Inflammation has been identified as an important factor in cancer development and progression in recent times.^8^,^9^ Prolonged systemic inflammatory condition has been shown to assist growth of cancer cells, increase vascularity of tumors and also cause distant metastasis leading to poor survival.^10^ In this context, literature is replete with markers which can predict survival in cancer patients like neutrophil-lymphocyte ratio, lymphocyte-to-monocyte ratio, platelet-lymphocyte ratio, C-reactive protein/albumin ratio, prognostic immune and nutritional index, Glasgow prognostic score, modified Glasgow prognostic Score, systemic immune-inflammation index (SII) and systemic immune response index (SIRI).^11^,^12^ However, which is the most reliable marker of these is a subject of debate.

SII^13^-^15^ and SIRI^16^-^18^ are both hematological markers which have found to be useful to predict prognosis after several cancers. SII can be calculated using: absolute platelet X neutrophil counts/ absolute lymphocyte count.^19^ While SIRI is calculated as: neutrophil count × monocyte count/lymphocyte count.^20^ The values required to calculate both SII and SIRI are easily available in any healthcare setup in the world and therefore both these markers can be readily applied in clinical practice. However, their value for cervical cancer is not clear as no systematic review has collated evidence from literature. We present the first review assessing if SII and SIRI can predict outcomes after cervical cancer.

## METHODOLOGY

This research work follows the PRISMA reporting guidelines.^21^ The protocol was registered on PROSPERO: CRD42024603478.

Specific inclusion criteria were established to ensure the relevance to the review. The eligibility of studies was guided by the well-defined framework based on population, interventions, comparisons and outcomes, as well as the study design. We chose to select all study designs conducted on cervical cancer patients (Population). Studies examining the relationship between high vs low SII or SIRI (Exposure and comparison) and Outcomes of cervical cancer namely, overall survival (OS) and disease-free survival (DFS) which was reported as either adjusted or unadjusted effect size were eligible. Studies published only as abstracts and which were not peer-reviewed were also ineligible. Outcomes were not predefined and all definitions were eligible.

CENTRAL library, Embase, PubMed, Scopus and Web of Science databases were searched for retrieving possible studies (from inception up to 25^th^ October 2024) by two independent reviewers. The search strategy was collaboratively developed by two co-authors using carefully selected free and MeSH words. Further details are in Supplementary Table I. During the search, we did not apply filters related to language or location or publication date. This query was repeated in all databases to ensure a detailed an unbiased search of studies. As an additional search, reference lists of included studies were also explored.

**Supplementary Table-I:** Search strategy

***PubMed***
1. (((cervical) AND (((cancer) OR (malignancy)) OR (carcinoma))) AND ((systemic immune inflammation index) OR (SII))
2. (((cervical) AND (((cancer) OR (malignancy)) OR (carcinoma))) AND ((Systemic immune response index) OR (SIRI))
***Embase***
#1: (‘cervical neoplasms’/exp OR ‘cervical cancer’ OR ‘cervical carcinoma’)
#2: (‘systemic AND immune AND inflammation AND index’ OR ‘SII’)
#3: (‘Systemic immune response index’ OR ‘SIRI’)
#4: #2 OR #3
#6: #1 AND #4
***Scopus***
1. (TITLE-ABS-KEY (cervical) AND (cancer) OR (malignancy) OR (carcinoma)) AND (TITLE-ABS-KEY (systemic immune inflammation index) OR (SII))
2. (TITLE-ABS-KEY (cervical) AND (cancer) OR (malignancy) OR (carcinoma)) AND (TITLE-ABS-KEY (Systemic immune response index) OR (SIRI))
***CENTRAL***
1. (ti,ab,kw (cervical) AND (cancer) OR (malignancy) OR (carcinoma)) AND (ti,ab,kw (systemic immune inflammation index) OR (SII))
2. (ti,ab,kw (cervical) AND (cancer) OR (malignancy) OR (carcinoma)) AND (ti,ab,kw (Systemic immune response index) OR (SIRI))
***Web of Science***
1. (((cervical) AND (((cancer) OR (malignancy)) OR (carcinoma))) AND ((systemic immune inflammation index) OR (SII))
2. (((cervical) AND (((cancer) OR (malignancy)) OR (carcinoma))) AND ((Systemic immune response index) OR (SIRI))

**Supplementary Table-II:** Risk of bias

Author	Selection of studies	Comparability	Outcome assessment	Adjusted outcomes	NOS score
Wang 2024	4	-	3	No	7
Staniewska 2024	4	2	3	Yes	9
Li 2024	4	2	2	Yes	8
Fullerton 2024	4	2	3	Yes	9
Bruno 2024	4	-	2	No	6
Shan 2023	4	2	2	Yes	8
Guo 2023	4	2	2	Yes	8
Ferioli 2023	4	-	2	No	6
Liu 2022*	4	2	3	Yes	9
Li 2021	4	2	2	Yes	8
Chao 2020	4	2	3	Yes	9
Huang 2019*	4	2	2	Yes	8
Holub 2019	4	-	2	No	6

NOS: Newcastle Ottawa scale

To avoid repeated screening of the same articles, we first deduplicated the search results electronically. The remaining studies were then examined by the two reviewers one by one by reading titles and abstracts only. Articles that did not meet the pre-established inclusion criteria were removed at this stage. All remaining studies were downloaded. Lastly, the full-texts were read by the same two reviewers. When both reviewers were satisfied, the study was included in the review. Any disagreements or uncertainties regarding the eligibility of articles were resolved through discussion. Risk of bias assessment was performed using the Newcastle-Ottawa scale (NOS). Herein too, both authors screened individual studies for the questions of NOS which assess the participant selection, comparability of groups and outcomes. Final scores were given after independent assessments by the reviewers which ranged from 0-9. Scores of 8-9 meant high, 6-7 meant medium, and <6 meant low quality. Disagreements were resolved by consensus.

Data extraction was conducted systematically to obtain information on author name, publication year, study type, cancer stage, histology, lymph node metastasis, management, sample size, age, type of marker, its cut-off, technique to assess the cut-off, follow-up and outcomes. Two authors performed the data extraction separately to minimize bias. It was further cross-checked to minimize errors.

Meta-analyses were performed in a random-effects model on “Review Manager” (RevMan, version 5.3). Preferably, adjusted outcomes were extracted from studies for analysis. However, if the study failed to report adjusted data, univariate analysis was extracted and it was marked in the data information sheet. The outcomes were then combined to determine pooled hazard ratio (HR) with 95% CI for both OS and DFS. We also quantified the inter-study heterogeneity using the I^2^ index of the software. Values over 50% indicated substantial heterogeneity. A sensitivity analysis was performed. Subgroup analysis was conducted based on country of the study, sample size, cancer stage, treatment, cut-off, use of adjusted data and NOS score. This was done only for OS and not DFS owing to small number of studies for the later analysis.

## RESULTS

**Fig.1:**
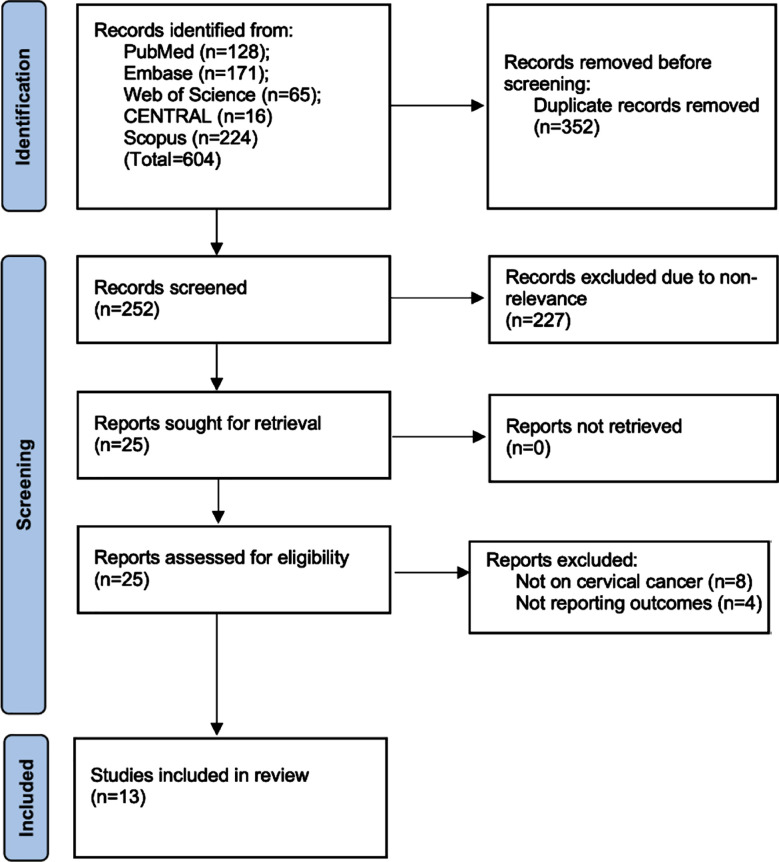
PRISMA flowchart of the review.

Thirteen studies met the inclusion criteria (Fig.1).^22^-^34^ Two studies^30^,^33^ included two cohorts each, therefore the total number of cohorts was 15. All were retrospective cohort studies including a total of 3030 cervical cancer patients (Table-I). Six studies included patients with stage I-II cancer while four studies included I-IV patients. In most studies’ majority patients had squamous cell carcinoma. Five studies treated patients only by chemoradiotherapy. In the remaining studies, patients underwent surgical intervention with or without chemotherapy/radiotherapy. Four studies examined both SII and SIRI while six studies were on SII and three were only on SIRI. All scores were calculated pretreatment. Likewise, follow-up ranged from 36 to 98 months. Four studies reported unadjusted data while remaining reported multivariate adjusted HR. On assessment of study quality, four studies were given a score of 6 or 7 while the remaining studies got a score of 8-9.

**Table-I:** Data obtained from studies.

Author	Design	Country	Sample size	Age (years)	FIGO Stage	SCC (%)	Node + (%)	Treatment	Marker	Cut-off	Method for cut-off	Follow-up (months)	Out-comes	Adjusted outcomes
Wang 2024	R	China	325	50	I-III	NR	NR	Surgery, Radiotherapy	SII SIRI	385.8 0.36	Youden index	50	OS	No
Staniewska 2024	R	Poland	249	57.2	I-IV	95.2	47.4	Chemo-radiotherapy	SII	986.01	ROC curve	75.8	OS	Yes
Li 2024	R	China	212	58.8	I-IV	91	50	Chemo-radiotherapy	SII	828	ROC curve	47	OS, DFS	Yes
Fullerton 2024	R	Canada	196	51	I-IV	84	36	Chemo-radiotherapy	SII	700	ROC curve	84	OS, DFS	Yes
Bruno 2024	R	Italy	174	47	I-II	55.2	30.5	Surgery +/- Chemo-radiotherapy	SII SIRI	663 0.98	ROC curve	53	OS, DFS	No
Shan 2023	R	China	72	51	I-II	91.7	5.6	Surgery + radiotherapy	SIRI	1.185	ROC curve	56.5	OS, DFS	Yes
Guo 2023	R	China	109	53.9	I-II	100	13.8	Surgery	SII SIRI	566.23 1.38	ROC curve	NR	OS, DFS	Yes
Ferioli 2023	R	Italy	173	56	I-IV	85	NR	Chemo-radiotherapy	SII SIRI	NR	NR	36	OS, DFS	No
Liu 2022*	R	China	133 77	51 48	I-II I-II	100 100	NR	Surgery + Chemotherapy	SII	600.56	ROC curve	98 84.6	OS, DFS	Yes
Li 2021	R	China	260	51	II	94.2	23.5	Chemo-radiotherapy	SIRI	1.02	ROC curve	47.3	OS, DFS	Yes
Chao 2020	R	China	441	42	I-II	NR	NR	Surgery +/- Chemo-radiotherapy	SIRI	1.25	Youden index	67	OS	Yes
Huang 2019*	R	China	328 130	45 44	I-II I-II	100 100	21.6 26.2	Surgery	SII	475	ROC curve	47 47	OS	Yes
Holub 2019	R	Spain	151	52.8	I-IV	76.8	NR	Surgery, chemotherapy, radiotherapy	SII	1000	ROC curve	43.8	OS	No

* Study had two cohorts

R, retrospective; FIGO, Fédération Internationale de Gynécologie et d’Obstétrique; SCC, squamous cell carcinoma; OS, overall survival; Disease free survival; ROC, receiver operating characteristic; NR, not reported; SII, systemic immune inflammation index; SIRI, systemic immune response index

### SII:

**Fig.2:**
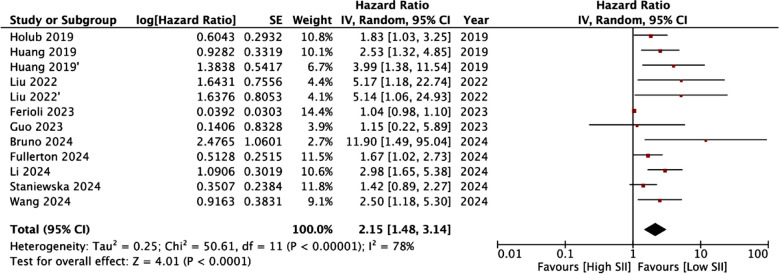
Forest plot of the analysis demonstrating association between SII and OS. IV, inverse variance; CI, confidence intervals; SE, standard error

**Fig.3:**
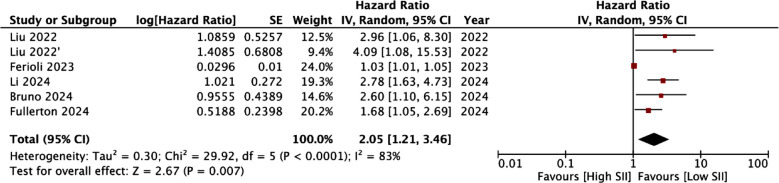
Forest plot of the analysis demonstrating association between SII and DFS. IV, inverse variance; CI, confidence intervals; SE, standard error

Ten studies with 12 cohorts reported adequate data for a meta-analysis for the association between SII and OS. We noted that high SII scores were associated with statistically significant increased risk of poor OS (HR: 2.15 95% CI: 1.48, 3.14 I^2^=78%) (Fig.2). The results were found to be robust on sensitivity analysis. Subgroup analysis results are shown in Table-II. The association between SII was significant for almost all subgroups except for studies with low NOS score, reporting unadjusted data and using chemoradiotherapy alone. Only five studies with six cohorts reported the association between SII and DFS. Meta-analysis showed that SII was a significant predictor for DFS in cervical cancer patients (HR: 2.05 95% CI: 1.21, 3.46 I^2^=83%) (Fig.3). These results also failed to change in significance during sensitivity analysis.

**Table-II:** Outcomes of subgroup analysis based on various baseline variables.

Factor	Groups	Number of cohorts	HR [95% CI]	I^2^
***SII-OS***				
Location	Chinese	7	2.87 [2.06, 4.00]	0
	Western	5	1.48 [1.01, 2.17]	71
Sample size	>200	4	2.16 [1.50, 3.13]	34
	<200	8	2.12 [1.28, 3.51]	74
Stage	I-IV	5	1.59 [1.07, 2.37]	80
	I-II	6	3.18 [2.01, 5.04]	0
Treatment	Chemoradiotherapy	4	1.55 [0.99, 2.43]	82
	Surgery +/- chemoradiotherapy	7	2.98 [2.01, 4.41]	0
Cut-off	≥700	4	1.83 [1.36, 2.47]	22
	<700	7	2.98 [2.01, 4.41]	0
Adjusted data	Yes	8	2.21 [1.60, 3.04]	28
	No	4	1.85 [0.97, 3.54]	79
NOS score	8-9	8	2.21 [1.60, 3.04]	28
	6-7	4	1.85 [0.97, 3.54]	79
***SIRI -OS***				
Location	Chinese	5	1.68 [1.28, 2.21]	0
	Western	2	1.08 [0.67, 1.75]	23
Sample size	>200	3	1.62 [1.22, 2.15]	0
	<200	4	1.49 [0.80, 2.79]	43
Stage	I-IV	1	0.99 [0.96, 1.02]	-
	I-II	5	1.71 [1.30, 2.26]	0
Treatment	Chemoradiotherapy	2	1.00 [0.90, 1.11]	8
	Surgery +/- chemoradiotherapy	4	2.02 [1.43, 2.84]	0
Cut-off	≥1	4	1.70 [1.28, 2.25]	0
	<1	2	1.70 [0.76, 3.82]	0
Adjusted data	Yes	4	1.70 [1.28, 2.25]	0
	No	3	0.99 [0.96, 1.02]	0
NOS score	8-9	4	1.70 [1.28, 2.25]	0
	6-7	3	0.99 [0.96, 1.02]	0

HR, hazard ratio; CI, confidence intervals; NOS, Newcastle Ottawa scale; SII, systemic immune inflammation index; SIRI, systemic immune response index; OS, overall survival

### SIRI:

**Fig.4:**
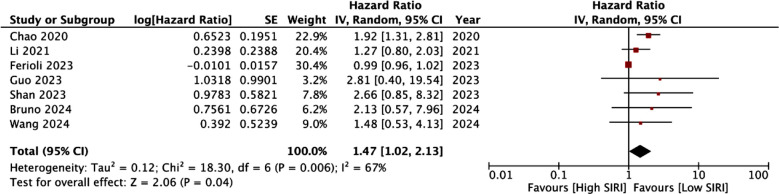
Forest plot of the analysis demonstrating association between SIRI and OS. IV, inverse variance; CI, confidence intervals; SE, standard error

There were seven studies examining the link between SIRI and OS after cervical cancer. Meta-analysis showed a statistically significant association between the two (HR: 1.47 95% CI: 1.02, 2.13 I^2^=67%) (Fig-4). However, the results were not stable on sensitivity analysis and became non-significant on exclusion of multiple studies. Results of subgroup analysis can be found in Table-II. Non-significant results were noted for one of the subgroups for all variables. No significant association was noted between SIRI and OS for studies on Western population, sample size <200, stage I-IV cancer, using chemoradiotherapy alone, with cut-off <1, reporting non-adjusted data and with a low NOS score.

**Fig.5:**
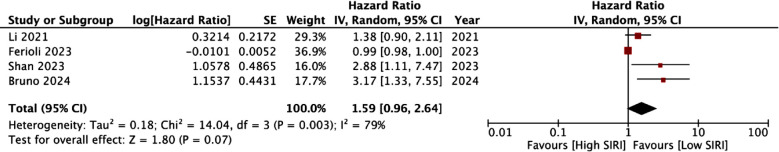
Forest plot of the analysis demonstrating association between SIRI and DFS. IV, inverse variance; CI, confidence intervals; SE, standard error

Only four studies reported the association between SIRI and DFS. Pooled analysis showed that SIRI did not predict DFS in cervical cancer patients (HR: 1.59 95% CI: 0.96, 2.64 I^2^=79%) (Fig.5). During sensitivity analysis, exclusion of the study of Ferioli et al^29^ changed the significance of the results denoting a positive association between SIRI and DFS (HR: 2.07 95% CI: 1.14, 3.75 I^2^=51%).

## DISCUSSION

We have conducted the first systematic review and meta-analysis of literature examining the ability of SII and SIRI to predict OS and DFS in cervical cancer patients. A detailed search of five commonly used literature databases along with gray literature revealed 13 studies with 15 cohorts examining the clinical query. After collation of coherent data, the meta-analysis showed that pre-treatment high SII scores were significantly associated with poor OS and DFS after cervical cancer. To be specific, high SII led to a two-fold increase in the risk of worse OS and DFS. Indeed, there were variations among the results of included studies with some denoting stronger association while others suggesting minimal connection between the two.

Nevertheless, the sensitivity analysis showed that no study had an undue influence on the results as there was no change in significance of the pooled HR on exclusion of any study. Lack of publication bias is another factor complementing the robustness of the outcomes. In contrast, the association between SIRI and outcomes of cervical cancer was not as strong as SII. The meta-analysis showed that high pre-treatment SIRI led to a 47% increase in the risk of poor OS after cervical cancer but no significant relationship was noted between SIRI and DFS. A major limitation of these results was the lack of robustness on sensitivity analysis. Most of the individual studies included in the meta-analysis of OS found no significant association with SIRI. Exclusion of individual studies showed non-significant results. Furthermore, the range of 95% CI was such that the heightened risk of worse OS ranged from just 2% to 113%. The smaller number of studies examining SIRI could be one factor contributing to such results and therefore the outcomes should be interpreted with caution.

A plethora of factors can influence survival in cases of cervical cancer. Important ones being age of diagnosis, cancer stage, treatment and comorbidities.^6^ One can note that there was significant heterogeneity in all our meta-analyses and one contributor to this could be the wide variation in the patient characteristics, cancer stage and treatment protocols between the studies. We therefore thoroughly explored these factors by means of a subgroup analysis. Firstly, it was noted that SII and SIRI had a stronger association with OS in Chinese studies as compared to Western studies. This could be related to the ethnic difference between Asians and Western populations as well as differences in organized screening and vaccination programs between the two regions. Ineffective surveillance in resource limited countries often leads to late diagnosis leading to lower survival.^35^

We also noted that studies reporting unadjusted data did not find any significant associations between SII/SIRI and OS but significant results were found with studies reporting multivariate adjusted data. This is reassuring since adjusted data provides better evidence as compared to unadjusted outcomes by taking into account potential confounding factors. Subgroup analysis based on treatment also showed non-significant results for studies on chemoradiotherapy alone but significant results for studies using surgical intervention. One reason for this anomaly could be the limited data for the former subgroup. Furthermore, the pooled HR was still >1 and lower end of 95% CI very close to One indicating a potential role of both markers for cervical cancer patients treated with chemoradiotherapy alone.

We also found non-significant results for studies using a lower cut-off of SIRI but no such difference was noted for SII. The stronger association between SII and OS and the larger number of studies available for this marker could be possible reasons for such results. However, the wide variations in the cut-offs used by the studies for both markers is a cause of concern. There is no definitive cut-off reported for both SII and SIRI in literature and most studies use population-specific markers for predicting survival.^13^-^15^ We therefore recommend that clinicians incorporating either SII or SIRI in their practice should calculate the appropriate cut-off for their cohort to best optimize the results.

The current review is in agreement with prior published literature examining the link between SII and SIRI and oncology patients. A pooled analysis of six studies shows that SII can be used to predict OS and progression-free survival (PFS) in patients with nasopharyngeal carcinoma.^36^ Another meta-analysis by Cheng et al^37^ has found that pre-treatment increased SII is independently associated with poor OS and DFS in breast cancer patients. Similar results have been shown in patients with gastric cancer^38^ and urinary tract cancers.^39^ A large number of studies have also examined the utility of SII for other gynecological cancers which have been systematically reviewed by other authors. For example, Ji et al^40^ noted SII to be an independent marker predicting both OS and PFS in patients with endometrial cancer. Mao et al^41^ combined outcomes of six studies to demonstrate the prognostic ability of SII in ovarian cancer.

Reviews have also validated the prognostic ability of SIRI for cancer patients, albeit with somewhat limited data as compared to SII. Wang et al^42^ collated data from nine studies to demonstrate that SIRI is a predictor of worse survival outcomes after nasopharyngeal carcinoma. Likewise, another review has validated its prognostic ability for oral cancer.^43^ A meta-analysis by Liang et al^44^ noted SIRI can predict OS and DFS for various gastrointestinal malignancies irrespective of tumor types, tumor stages and primary treatments. On the other hand, literature is scarce for the association between SIRI and gynecological cancers. To the best of our knowledge only Feng et al^45^ in a study of 118 patients have shown that SIRI is a predictor of five years OS in ovarian cancer. Except for the cervical cancer studies included in this review, there have been no other studies examining the association between SIRI and survival after gynecological cancers.

The prognostic potential of SII and SIRI can be attributed to the individual hematological components used by these indices namely, neutrophils, lymphocytes and platelets (SII) or monocytes (SIRI). High neutrophil count which can raise both SII and SIRI is associated with release of pro-inflammatory cytokines like interleukins, vascular endothelial growth factor and chemokines leading to a chronic inflammatory state. This in-turn causes increased tumor invasion, tumor vascularity and metastasis. Neutrophils also increase the levels of reactive oxygen species, arginase and nitric oxide which inhibits T-lymphocytes and therefore cancer-immunity.^46^

The role of platelets in promoting tumor growth has also been supported by literature. These cells improve epithelial-mesenchymal transition of tumor cells leading to better motility, improved tumor cell extravasation and high resistance to apoptosis.^47^,^48^ Monocytes also secrete high levels of chemokines and cytokines which promotes cancer growth.^49^ Differentiation into tumor-associated macrophages leads to CD8+ T cell death. These cells act as a defense against tumor cells and decreased counts lead to tumor progression.^50^ The common denominator in both SII and SIRI is the lymphocyte count which are inversely related to tumor growth. Indeed, it is well recognized that lymphocytes act against cancer cells leading to reduced progression. T-lymphocytes directly attack cancer cells while B lymphocytes act via cytokines like interferon-gamma and tumor necrosis factor-alpha. Lastly, natural killer cells also act against cancer cells bypassing the antigen pathway.^8^ It can be noted that the components of SII and SIRI represent the baseline inflammatory and immune status of a cancer patient. Given the strong association between inflammation and cancer progression,^8^,^9^ these can therefore be reliable markers for predicting survival after cervical cancer.

### Strength of the review:

Being the first systematic review, it provides pooled evidence on the prognostic ability of SII and SIRI for cervical cancer. We also segregated outcomes based on several subgroups to allow the better interpretation of evidence. Given the easy availability of baseline values to calculate SII and SIRI, we believe that SII and SIRI can be easily incorporated in clinical practice to allow rapid bed-side prognostication of cervical cancer patients.

### Limitations:

Variations in the study populations, cancer stage and treatment are one of the prominent limitations of the review and also a major contributor to the inter-study heterogeneity. Further, the retrospective study designs of all articles lower the quality of evidence. Another factor limiting the interpretation of the results is the lack of optimal cut-off for both SII and SIRI. The small number of studies, several with limited sample size is another cause of concern. The included studies also did not uniformly report data on DFS which reduced the power of the meta-analysis. For the same reason, a subgroup analysis could not be conducted for DFS. Next, not all studies reported adjusted data and there could have been known and unknown confounders influencing the results. We purposely included unadjusted data in the review so that the present study provides the most comprehensive evidence on the role of SII and SIRI for cervical cancer. Lastly, evidence was generated by analysis of studies from a few selected populations. A large number of studies from various ethnicities are needed to allow generalization of results.

## CONCLUSIONS

High SII values can be predictive of poor OS and DFS in patients with cervical cancer. Likewise, high SIRI was also found to predict worse OS but not DFS. The association was found to be more robust for SII rather than SIRI. Future prospective studies on different ethnic populations need to be planned to complement the current results.

### Authors’ contributions:

**FJ:** Study design , literature search and manuscript writing.

**FJ and PZ:** Data collection, data analysis and interpretation. Critical Review.

**FJ:** Manuscript revision and validation and is responsible for the integrity of the study. All authors have read and approved the final manuscript.
